# The pyrenoid: the eukaryotic CO_2_-concentrating organelle

**DOI:** 10.1093/plcell/koad157

**Published:** 2023-06-04

**Authors:** Shan He, Victoria L Crans, Martin C Jonikas

**Affiliations:** Department of Molecular Biology, Princeton University, Princeton, NJ 08540, USA; Howard Hughes Medical Institute, Princeton University, Princeton, NJ 08540, USA; Department of Molecular Biology, Princeton University, Princeton, NJ 08540, USA; Department of Molecular Biology, Princeton University, Princeton, NJ 08540, USA; Howard Hughes Medical Institute, Princeton University, Princeton, NJ 08540, USA

## Abstract

The pyrenoid is a phase-separated organelle that enhances photosynthetic carbon assimilation in most eukaryotic algae and the land plant hornwort lineage. Pyrenoids mediate approximately one-third of global CO_2_ fixation, and engineering a pyrenoid into C_3_ crops is predicted to boost CO_2_ uptake and increase yields. Pyrenoids enhance the activity of the CO_2_-fixing enzyme Rubisco by supplying it with concentrated CO_2_. All pyrenoids have a dense matrix of Rubisco associated with photosynthetic thylakoid membranes that are thought to supply concentrated CO_2_. Many pyrenoids are also surrounded by polysaccharide structures that may slow CO_2_ leakage. Phylogenetic analysis and pyrenoid morphological diversity support a convergent evolutionary origin for pyrenoids. Most of the molecular understanding of pyrenoids comes from the model green alga Chlamydomonas (*Chlamydomonas reinhardtii*). The Chlamydomonas pyrenoid exhibits multiple liquid-like behaviors, including internal mixing, division by fission, and dissolution and condensation in response to environmental cues and during the cell cycle. Pyrenoid assembly and function are induced by CO_2_ availability and light, and although transcriptional regulators have been identified, posttranslational regulation remains to be characterized. Here, we summarize the current knowledge of pyrenoid function, structure, components, and dynamic regulation in Chlamydomonas and extrapolate to pyrenoids in other species.

## Introduction

Photosynthesis forms the base of the food chain in most ecosystems by converting CO_2_ from the environment into organic carbon. At the heart of these reactions is the enzyme Ribulose-1,5-bisphosphate carboxylase/oxygenase (Rubisco), which assimilates CO_2_ into sugar precursors used to generate biomass (Bracher et al. 2017). Despite its crucial role in photosynthesis, Rubisco has two limitations: (1) it has a slow catalytic rate for an enzyme in central carbon metabolism; and (2) it can also catalyze oxygenation, a wasteful reaction that uses O_2_ instead of CO_2_ (Flamholz et al. 2019). A tradeoff between Rubisco's catalytic rate and specificity for CO_2_ over O_2_ appears to prevent the evolution or engineering of Rubisco to be both fast and specific (Tcherkez et al. 2006; Savir et al. 2010; Flamholz et al. 2019). To keep oxygenation at tolerably low levels, many plants use a specific but slow form of Rubisco and compensate for its slow catalytic rate by producing a large amount of the enzyme (Raven 2013). This strategy requires significant cellular resources, including up to 25% of total leaf nitrogen (Raven 2013).

Some photosynthetic organisms overcome the limitations of Rubisco by using a CO_2_-concentrating mechanism (CCM) to deliver concentrated CO_2_ to the enzyme. This concentrated CO_2_ increases the turnover rate of Rubisco, and the higher ratio of CO_2_ to O_2_ favors carboxylation and suppresses oxygenation (Badger et al. 1980; Kupriyanova et al. 2023). There is currently great interest in understanding how CCMs work, both because of their significant ecological role (Ehleringer et al. 1991; Badger et al. 2006; Meyer et al. 2017) and because engineering a CCM into crops has the potential to increase yields (Matsuoka et al. 2001; Kajala et al. 2011; Hanson et al. 2016; Hennacy and Jonikas 2020; Adler et al. 2022).

CCMs are categorized into two broad classes: biochemical and biophysical, depending on the nature of the intermediate molecules used to concentrate CO_2_. Biochemical CCMs, which include C_4_, C_2_, and crassulacean acid metabolism (CAM), transiently fix CO_2_ into intermediate organic molecules such as oxaloacetate and malate, from which concentrated CO_2_ is released in proximity to Rubisco (Caemmerer and Furbank 2003; Sage et al. 2012; Heyduk et al. 2019). By contrast, in biophysical CCMs, the only intermediate molecule is bicarbonate (HCO_3_^−^) (Hennacy and Jonikas 2020). Biochemical CCMs are predominantly found in plants and typically involve multicellular structures, whereas biophysical CCMs are predominantly found in microbes and operate at a single-cell level (Maberly and Gontero 2017).

Biophysical CCMs differ between prokaryotes and eukaryotes. Both rely on a subcellular structure whose matrix contains a high concentration of Rubisco, into which concentrated CO_2_ is released from HCO_3_^−^ (Wang et al. 2015; Kaplan 2017; Hennacy and Jonikas 2020; Adler et al. 2022; Ang et al. 2022). However, the eukaryotic compartment known as the pyrenoid (1-2 *µ*m in diameter) is much bigger than the bacterial Rubisco-containing compartment, the carboxysome (∼200 nm in diameter). Additionally, CO_2_ delivery to Rubisco in the two structures is thought to be achieved based on different principles: in pyrenoids, CO_2_ delivery is mediated by thylakoid membranes, as discussed below (Fei et al. 2022), whereas in carboxysomes, CO_2_ is produced from HCO_3_^−^ diffusing directly into the carboxysome matrix (Mangan et al. 2016). This review will focus on the pyrenoid.

**Figure 1.**
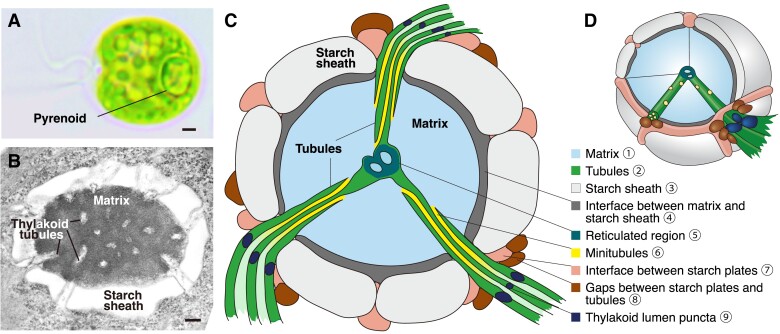
Structure of the Chlamydomonas pyrenoid. **A)** The Chlamydomonas pyrenoid is visible by light microscopy. Scale bar, 1 *μ*m. **B)** The Chlamydomonas pyrenoid is composed of three major compartments: the Rubisco matrix, thylakoid tubules, and starch sheath. Scale bar, 200 nm. **C)** Two-dimensional and **D)** Three-dimensional models of the pyrenoid showing the major compartments and protein peripheral structures. See Table 1 for a list of the known protein components of each structure. (The circled numbers indicating the sub-pyrenoid localizations in panel **C** are coordinated with the circled numbers in Table 1).

Pyrenoids are found inside the chloroplasts of most eukaryotic algae (including most microalgae and many macroalgae) and some species of nonvascular land plants called hornworts (Villarreal and Renner 2012; Meyer et al. 2017; Li et al. 2020). The pyrenoid is typically visible under light microscopy as a 1–2 *µ*m punctum within the chloroplast (Fig. 1A).

One of the earliest records of a pyrenoid dates from 1782 by the Danish naturalist and scientific illustrator Otto Frederik Müller, who drew unnamed puncta in sketches of the green alga *Spirogyra* (formerly *Conferva jugalis*) (Müller 1782), making it one of the first scientifically documented organelles. The pyrenoid was first described in a publication in 1803 (Vaucher 1803). The term pyrenoid was conceived in 1882 from the Greek πυρην (pyren, kernel) (Schmitz 1882). From this point on, the pyrenoid became the focus of many classic morphological studies using light and electron microscopy. Further reading on the history of pyrenoid research can be found in a recent review by Barrett et al. and a book chapter by Meyer et al. (Meyer et al. 2020a; Barrett et al. 2021).

Research on pyrenoids has recently gained momentum and currently has three major motivations: (1) a growing appreciation for the major role of pyrenoids in the global carbon cycle; (2) prospects to engineer pyrenoids into crops to increase yields; and (3) the unique value of the pyrenoid as a model for biological phase-separated condensates, a recently discovered ubiquitous class of organelles. We discuss each of these motivations below.

Pyrenoids play a major role in the global carbon cycle, mediating approximately 30% to 40% of global CO_2_ assimilation each year (Mackinder et al. 2016). Approximately one-half of global CO_2_ assimilation occurs in the oceans (Field et al. 1998; Behrenfeld et al. 2001), and most of this assimilation is attributed to eukaryotic algae (Flombaum et al. 2013; Rousseaux and Gregg 2013), nearly all of which have pyrenoids (Mann 1996; Not et al. 2004; Thierstein and Young 2004; Meyer and Griffiths 2013).

There is a growing interest in enhancing yields of major global crops that do not have CCMs by engineering a CCM into them (Rae et al. 2017; Hennacy and Jonikas 2020). Among the various CCMs that could be engineered, the green algal pyrenoid-based CCM is a particularly promising candidate for engineering into non-CCM plants as a result of two attractive qualities: (1) it operates at the single-cell level, which means that leaf anatomy does not need to be engineered as would be necessary for engineering of the C_4_ CCM (Kajala et al. 2011); and (2) unlike the prokaryotic carboxysome-based CCM, the green algal pyrenoid-based CCM is natively encoded in the eukaryotic nuclear genome, which could facilitate its engineering into monocot crops such as wheat (*Triticum aestivum*) and rice (*Oryza sativa*), whose prokaryotic chloroplast genomes remain challenging to engineer.

The pyrenoid is also of interest from a fundamental science perspective, as it is a phase-separated organelle (Freeman Rosenzweig et al. 2017). Biological phase separation underlies the formation of many cellular structures (Shin and Brangwynne 2017). The pyrenoid is one of the few phase-separated organelles where the functional value of condensate formation is understood, as there is a clear functional and fitness cost to preventing Rubisco condensation into a matrix (Meyer et al. 2012; Mackinder et al. 2016; He et al. 2020; Fei et al. 2022). Moreover, the pyrenoid of the model alga Chlamydomonas (*Chlamydomonas reinhardtii*) is one of the structurally best understood phase-separated condensates, as its phase separation was reconstituted in vitro (Wunder et al. 2018) and the structural basis behind this phase separation was determined (He et al. 2020), making it a powerful system for deriving the basic fundamental principles that underlie the assembly of phase-separated organelles.

The vast majority of our molecular understanding of pyrenoids comes from recent studies in Chlamydomonas. As a well-established model organism widely used for photosynthesis studies, Chlamydomonas benefits from a thriving community of researchers who have produced genome sequences and annotations (Merchant et al. 2007; Craig et al. 2023), genome-wide omics data (Brueggeman et al. 2012; Fang et al. 2012; Zones et al. 2015; Strenkert et al. 2019), mutant libraries (Li et al. 2016; Li et al. 2019), fluorescently tagged lines for gene functional analysis (Mackinder et al. 2017; Wang et al. 2022), as well as pyrenoid proteomes and a pyrenoid proxiome (Mackinder et al. 2017; Zhan et al. 2018; Lau et al. 2023). Such resources are currently lacking for other algal species, although recent progress has been made toward developing similar tools in model diatoms such as high-efficiency transformation protocols in *Phaeodactylum tricornutum* (Miyagawa et al. 2009), stably propagated episomes in *P. tricornutum* and *Thalassiosira pseudonana* (Karas et al. 2015), proteome analyses of mitochondria and plastids in *T. pseudonana* (Schober et al. 2019), and a fluorescent protein-tagging pipeline in *T. pseudonana* (Nam et al. 2022).

In this review, we discuss the basic concepts of pyrenoid function as well as the current understanding of pyrenoids in Chlamydomonas, other algae, and hornworts. Some aspects of these topics have been covered in recent reviews (Barrett et al. 2021; Adler et al. 2022). Our review seeks to provide an update on the most recent discoveries in the field and discuss the evolution, biogenesis, regulation, and function of the pyrenoid-based CCM.

## Operating principles and evolution

### Operating principles of the pyrenoid-based CCM

Photosynthetic cells can obtain their carbon from two sources in the environment: CO_2_ and HCO_3_^−^. The availability of each source can vary depending on the environment (e.g. aquatic growth or growth on surfaces exposed to air) and conditions (e.g. external pH). In the aquatic environment, HCO_3_^−^ is normally more abundant than CO_2_, whereas CO_2_ may be more available to cells growing on the surface of particles in the soil.

**Figure 2.**
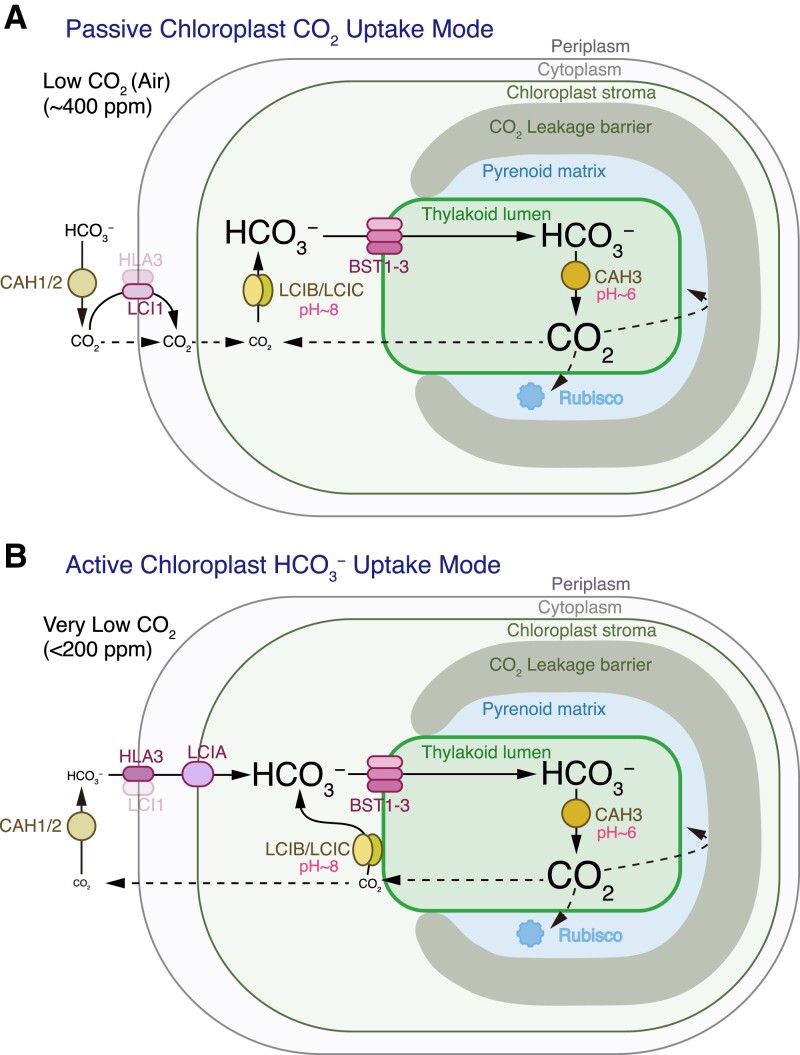
Operating principles of the pyrenoid-based CCM. The Chlamydomonas CO_2_-concentrating mechanism is shown; the basic principles are likely to apply in other species, although due to convergent evolution, the specific proteins that mediate some of the reactions may be phylogenetically unrelated to those in Chlamydomonas. Mutant phenotypes and biophysical modeling (Fei et al. 2022) support the existence of two operating modes of a pyrenoid-based CO_2_-concentrating mechanism, which differ based on how HCO_3_^−^ is accumulated in the chloroplast stroma. **A)** The first mode uses a passive chloroplast CO_2_ uptake strategy, where CO_2_ passively diffuses across the chloroplast envelope into the stroma and is converted into HCO_3_^−^ by the LCIB/LCIC carbonic anhydrase complex. This strategy is used under low CO_2_ (ambient air levels of external CO_2_). **B)** The second mode uses an active chloroplast HCO_3_^−^ uptake strategy, which relies on active pumping of HCO_3_^−^ into the chloroplast. This strategy is used under very low CO_2_.

CO_2_ is difficult to concentrate directly within cells because it is a small, uncharged molecule that rapidly leaks across membranes. Current models of the pyrenoid-based CCM suggest that, to overcome this issue, cells use carbonic anhydrases to convert CO_2_ to the charged molecule HCO_3_^−^, which cannot easily diffuse across membranes and can be directed to subcellular compartments via transmembrane transporters. Intercompartmental pH differences are thought to play a crucial role in this process by driving the interconversion of CO_2_ to HCO_3_^−^ in the appropriate cellular compartments (Fig. 2) (Hennacy and Jonikas 2020; Wang and Jonikas 2020; Fei et al. 2022).

A recent study (Fei et al. 2022) provides a detailed computational model of the Chlamydomonas CCM that is consistent with all available experimental evidence. We expect that the model will also be generally relevant to other algae, as most algae face similar biophysical challenges and the model is robust over broad parameter ranges.

Interestingly, this model indicates that two distinct CCM operating modes are feasible, which share a common core but differ in how HCO_3_^−^ is accumulated in the chloroplast stroma (Fei et al. 2022) (Fig. 2). At the common core of the two operating modes, stromal HCO_3_^−^ is transported into the thylakoid lumen, likely by the bestrophin-like channels BST1 (encoded by Cre16.g662600), BST2 (Cre16.g663400), and/or BST3 (Cre16.g663450), although the role of each BST remains unclear (Mukherjee et al. 2019). Inside specialized pyrenoid-traversing regions of the thylakoid membranes, carbonic anhydrase 3 (CAH3, Cre09.g415700) converts HCO_3_^−^ to CO_2_, which is driven by the low pH of the thylakoid lumen (Karlsson et al. 1998; Hanson et al. 2003; Blanco-Rivero et al. 2012; Burlacot et al. 2022). This CO_2_ diffuses out of the thylakoid membranes and into the pyrenoid matrix, where it is captured by Rubisco. A CO_2_ leakage barrier is thought to slow the escape of CO_2_ from the pyrenoid, increasing CO_2_ concentration and decreasing energetic costs (Fei et al. 2022). In the case of Chlamydomonas, modeling and experimental evidence suggest that the starch sheath (Toyokawa et al. 2020; Fei et al. 2022) and thylakoid membrane sheets (Fridlyand 1997; Fei et al. 2022) can serve as CO_2_ leakage barriers.

The two pyrenoid-based CCM operating modes use different strategies to accumulate HCO_3_^−^ in the chloroplast stroma, as follows. The first mode uses a passive chloroplast CO_2_ uptake strategy, where CO_2_ passively diffuses from the periplasm across the plasma membrane via the channel low CO_2_-inducible 1 (LCI1, Cre03.g162800) (Ohnishi et al. 2010; Kono and Spalding 2020) and across the chloroplast envelope into the chloroplast stroma (Fig. 2A). CO_2_ diffusing into the chloroplast or leaking out of the pyrenoid is converted to HCO_3_^−^ by the low-CO_2_-inducible B/C (LCIB/LCIC, Cre10.g452800/Cre06.g307500) carbonic anhydrase complex in a reaction driven by the high pH in the chloroplast stroma (Wang and Spalding 2006; Yamano et al. 2010; Jin et al. 2016; Kasili et al. 2023).

By contrast, the second CCM operating mode uses an active chloroplast HCO_3_^−^ uptake strategy. CO_2_ is converted to HCO_3_^−^ at the periplasm by the carbonic anhydrases CAH1 (Cre04.g223100) and CAH2 (Cre04.g223050) (Fujiwara et al. 1990; Van and Spalding 1999). HCO_3_^−^ crosses the plasma membrane via the transporter high light activated 3 (HLA3, Cre02.g097800) and is then concentrated across the chloroplast envelope by LCIA (Cre06.g309000), which in this model is an active HCO_3_^−^ pump (Fig. 2B) (Miura et al. 2004; Yamano et al. 2015). We note that LCIA has not been experimentally shown to actively pump HCO_3_^−^ across a membrane, but active pumping seems likely as the model indicates that passive HCO_3_^−^ channels across the chloroplast envelope fail to achieve an effective CCM (Fei et al. 2022). CO_2_ leaking out of the pyrenoid is recaptured by the LCIB/LCIC carbonic anhydrase complex, which relocalizes to the periphery of the pyrenoid (Wang and Spalding 2014a) to enhance the efficiency of CO_2_ recapture and avoid conversion of HCO_3_^−^ to CO_2_ near the chloroplast envelope, which would lead to loss of accumulated chloroplast HCO_3_^−^ (Fig. 2B) (Fei et al. 2022).

The passive CO_2_ uptake strategy and active HCO_3_^−^ uptake strategy have different performance depending on external CO_2_ concentrations and pH (Fei et al. 2022). The passive CO_2_ uptake strategy is effective and energetically efficient under ambient air levels of external CO_2_ (0.04%, 400 ppm; equivalent to 10 *μ*M cytosolic in the model) but is unable to deliver enough CO_2_ to saturate Rubisco under lower levels of CO_2_ (0.004%, also known as “very low CO_2_”; corresponding to 1 *μ*M cytosolic CO_2_ in the model). Accordingly, Chlamydomonas appears to use the passive CO_2_ uptake strategy under air levels of external CO_2_ but not under very low CO_2_, as evidenced by the severe growth defects of the *lcib* mutant under air levels of CO_2_ but not very low CO_2_ (Wang and Spalding 2006, 2014a, 2014b; Duanmu et al. 2009; Kono and Spalding 2020). In contrast to the passive CO_2_ uptake strategy, modeling suggests that the active HCO_3_^−^ uptake strategy can be effective and energetically efficient under both growth conditions (Fei et al. 2022). Intriguingly, despite the predicted good performance of the active HCO_3_^−^ uptake strategy under air levels of CO_2_ in silico, in vivo Chlamydomonas appears to reserve this strategy only for very low CO_2_ conditions, as evidenced by O_2_ evolution experiments (Wang and Spalding 2014a; Yamano et al. 2015; Kono and Spalding 2020). A possible explanation for this observation is that when Chlamydomonas is grown under air level CO_2_ conditions, the active HCO_3_^−^ uptake strategy may incur additional energetic costs beyond those accounted for in the model. Indeed, the model only considered energetic costs in the chloroplast, and it is possible that under certain conditions the active HCO_3_^−^ uptake strategy requires energetic input outside the chloroplast, for example to pump HCO_3_^−^ across the plasma membrane.

The operation of a pyrenoid-based CCM under either air or very low CO_2_ is estimated to be feasible for as little as the energetic equivalent of approximately 1 ATP per CO_2_ fixed (Fei et al. 2022), making it energetically inexpensive relative to the overall cost of CO_2_ fixation by the Calvin-Benson-Bassham cycle, which is approximately energetically equivalent to 9 ATPs per CO_2_ fixed (Mangan et al. 2016).

The energy for operating the CCM must ultimately come from the light reactions, which directly drive the pH difference between the thylakoid lumen and stroma and indirectly maintain the pH of other compartments and drive the activities of transporters. A recent study suggested that the protons needed to drive conversion of HCO_3_^−^ to CO_2_ in the thylakoid lumen are produced by photosynthetic cyclic electron flow mediated by proton gradient regulation-like 1 (PGRL1, Cre07.g340200) and pseudocyclic electron flow resulting from O_2_ photoreduction mediated by flavodiiron proteins (FLVs, Cre12.g531900 and Cre16.g691800) (Burlacot et al. 2022). The same study also found that chloroplast-to-mitochondria electron flow contributes to energizing the CCM, potentially by supplying ATP to drive transporters.

Rubisco fixes CO_2_ through carboxylation of ribulose-1,5-bisphosphate (RuBP) to produce 3-phosphoglycerate (3-PG or 3-PGA). While some 3-PG goes on to other parts of metabolism, most of it is metabolized in the Calvin-Benson-Bassham cycle to regenerate RuBP, allowing the cycle to continue (Calvin 1962). Interestingly, in Chlamydomonas, Rubisco is the only enzyme of the Calvin-Benson-Bassham cycle found in the pyrenoid; all other Calvin-Benson-Bassham cycle enzymes and associated regulatory proteins that have been localized are enriched in a region of the stroma immediately surrounding the pyrenoid (Fig. 1) (Küken et al. 2018; Wang et al. 2022). Thus, the Rubisco substrate RuBP and its product 3-PG need to exchange efficiently between the stroma and the pyrenoid. The pathway and mechanism of this exchange are currently unknown in any organism with a pyrenoid.

### Pyrenoids are likely the product of convergent evolution

**Figure 3.**
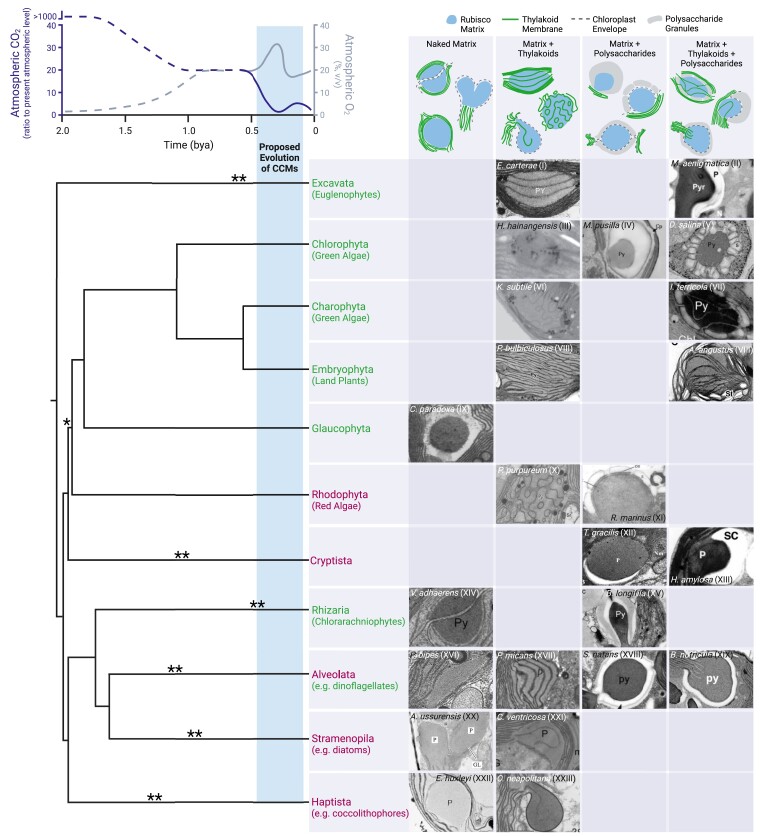
Pyrenoids appear to have convergently evolved in response to declining atmospheric CO_2_ levels. Approximate CO_2_ and O_2_ concentrations over time (Berner 2006; Whitney et al. 2011) are correlated with the phylogenetic tree of photosynthetic eukaryotes below (Strassert et al. 2021). Branch points correlate with the approximate timing of the divergence of different groups (see Strassert et al. 2021 and Bowles et al. 2022 for discussions on uncertainties regarding branch points). Asterisks denote the approximate timing of the acquisition of plastids through primary (*) or secondary (**) endosymbiosis (Jackson et al. 2018; Strassert et al. 2021). The blue shade highlights the proposed range for the timing of CCM evolution in different photosynthetic species (Villarreal and Renner 2012; Meyer et al. 2020a). Green and red lineages are denoted as green or purple text, respectively (most dinoflagellates have red plastids with the exception of *Lepidodinium* sp., which have green plastids) (Kamikawa et al. 2015). Representative electron micrographs of pyrenoids are shown below cartoons of four general pyrenoid types, displaying the wide variety of morphologies observed in each algal lineage and the hornworts. Roman numerals on the electron micrographs denote references to their original publications as follows: (I) Kusel-Fetzmann and Weidinger 2008; (II) Nudelman et al. 2006; (III) Zhang et al. 2008; (IV) van Baren et al. 2016; (V) Borowitzka 2018; (VI) Goudet et al. 2020; (VII) Mikhailyuk et al. 2014; (VIII) Duff et al. 2007; (IX) Hall and Claus 1963; (X) Nelson and Ryan 1988; (XI) Ford 1984; (XII) Laza-Martínez et al. 2012; (XIII) Clay and Kugrens 1999; (XIV) Shiratori et al. 2017; (XV) Ota et al. 2007; (XVI) Schnepf and ElbräChter 1999; (XVII) Kowallik 1969; (XVIII) Hansen and Daugbjerg 2009; (XIX) Decelle et al. 2021; (XX) Bedoshvili et al. 2009; (XXI) Bedoshvili and Likhoshway 2012; (XXII) Buma et al. 2000; (XXIII) Fresnel and Probert 2005. This figure was created with BioRender.

The predominant theory regarding the origins of pyrenoids is based on historical changes in atmospheric CO_2_ and O_2_ concentrations and the evolution of polyphyletic algal lineages. Oxygenic photosynthesis first evolved in cyanobacteria approximately 3 billion years ago (bya) (Schirrmeister et al. 2015) at a time when atmospheric CO_2_ concentrations were high and O_2_ concentrations were low (Fig. 3). The advent of oxygenic photosynthesis led to the Great Oxidation Event approximately 2.4 bya, when atmospheric O_2_ concentrations first began to rise (Anbar et al. 2007). Eukaryotic algae are thought to have first evolved approximately 1.5 to 2.0 bya (Yoon et al. 2004; Sánchez-Baracaldo et al. 2017; Strassert et al. 2021), a time when the atmospheric CO_2_:O_2_ ratio was still high and CCMs were likely not necessary for efficient growth (Raven et al. 2017). Over the course of approximately the next billion years, a diverse set of algal lineages arose through a complex series of endosymbiotic events (Falkowski et al. 2004; Reyes-Prieto et al. 2007; Keeling 2010; Dorrell et al. 2017; Jackson et al. 2018; Strassert et al. 2021). These different lineages can be split into two broad groups, green lineage algae and red lineage algae, which are distinguished by their use of different chlorophyll accessory pigments (Falkowski et al. 2004; Keeling 2010).

During the time that these algal lineages were evolving, CO_2_ concentrations were trending downward (Berner and Kothavala 2001; Berner 2006), a phenomenon accelerated by the evolution of land plants between 500 and 360 million years ago (mya), after each algal lineage had already been established (Fig. 3) (Berner 1997; Morris et al. 2018). This decrease in atmospheric CO_2_ and the simultaneous increase in atmospheric O_2_ are thought to be the main driving forces for the evolution of CCMs in aquatic microorganisms, leading to the theory that pyrenoids and other CCMs evolved independently via convergent evolution (Villarreal and Renner 2012; Rae et al. 2013; Raven et al. 2017; Meyer et al. 2020a).

This convergent evolution theory potentially explains why pyrenoids first evolved, but it remains difficult to pinpoint the exact timing of their origin. In the absence of concrete evidence from the fossil record (Knoll 1992), previous reviews have estimated the most likely timeline for CCM evolution by considering how various factors—including fluctuating CO_2_ and O_2_ concentrations, temperature changes, nutrient levels, and the kinetic properties of different forms of Rubisco—would have influenced the growth advantage conferred by a CCM (Griffiths et al. 2017; Raven et al. 2017; Meyer et al. 2020a). These reviews estimate that CCMs may have evolved in cyanobacteria and algae approximately 300 to 450 mya (Badger and Price 2003; Griffiths et al. 2017), likely when the atmospheric CO_2_ concentration was 2 to 16 times the present level (Raven et al. 2017). In hornworts (discussed further below), pyrenoids are estimated to have evolved approximately 100 mya (Villarreal and Renner 2012). These estimates all correspond to a time when different photosynthetic lineages were already established and support the convergent evolution theory.

The convergent evolution theory can be tested by comparing the sequences of proteins thought to perform the same functions in the pyrenoids of phylogenetically distant algal species, but this is currently difficult to do because the molecular composition of most pyrenoids is unknown. There are, however, three lines of molecular evidence that support the convergent evolution theory. The first is that thylakoid-luminal carbonic anhydrases of different types are necessary for CCM function in different lineages: the Chlamydomonas CCM requires the alpha-type carbonic anhydrase CAH3 (Karlsson et al. 1998; Hanson et al. 2003; Sinetova et al. 2012), whereas the diatom *P. tricornutum* requires a theta-type carbonic anhydrase (Kikutani et al. 2016; Matsuda et al. 2017).

The second piece of molecular evidence that supports convergent evolution is based on the different forms of Rubisco across lineages. There are at least four distinct types of Rubisco enzymes within algae and cyanobacteria, which differ greatly in their kinetic properties and holoenzyme structure (Badger et al. 1998). The fact that pyrenoids in different lineages package vastly different Rubisco holoenzymes supports the theory that they evolved convergently.

The third piece of evidence is related to the Chlamydomonas protein Essential Pyrenoid Component 1 (EPYC1, Cre10.g436550, also known as LCI5) (Turkina et al. 2006; Mackinder et al. 2016), which is a linker protein that clusters Rubisco together to form the pyrenoid matrix (Mackinder et al. 2016; He et al. 2020). EPYC1 is necessary for the Chlamydomonas CCM, but no homologs of this protein could be identified in algae beyond the closely related Volvocales (Mackinder et al. 2016), suggesting that its function is performed by other proteins that may have convergently evolved in different algal species. Repeat proteins with similar predicted properties as EPYC1 have been identified in other algae (Mackinder et al. 2016), and work is ongoing to characterize these and other putative linker proteins.

In addition to research on the algal CCM, several studies have been conducted on the evolution of CCMs in hornworts, which are the only land plants known to have pyrenoids. Hornworts are nonvascular plants thought to have been important in the water-to-land transition during embryophyte evolution (Qiu et al. 2006). The first hornwort pyrenoids evolved approximately 100 mya (Villarreal and Renner 2012), coinciding with a drastic decline in atmospheric CO_2_ levels (Fig. 3). The presence of a pyrenoid in hornworts is correlated with CCM activity detected by organic isotope discrimination and mass spectrometry analyses (Smith and Griffiths 1996a, 1996b, 2000; Hanson et al. 2002; Meyer et al. 2008), suggesting that pyrenoids play a similar role in hornwort CCMs as they do in algal CCMs. Phylogenetic evidence and ultrastructural data suggest that hornwort pyrenoids were gained and lost 5 to 6 times independently since they first appeared (Villarreal and Renner 2012). Interestingly, the distribution of pyrenoids across the green lineage of algae also suggests that multiple independent losses and gains have occurred (Meyer and Griffiths 2013). The environmental factors favoring pyrenoid loss remain unclear.

## Structure and components

Pyrenoid morphology can vary greatly depending on the species (Fig. 3), but the one unifying feature of all pyrenoids is the Rubisco matrix, which contains densely packed Rubisco (Holdsworth 1971; Borkhsenious et al. 1998). Most algae have one matrix per cell, although some species have multiple Rubisco matrices that can vary in size and shape (Meyer et al. 2020a). The Rubisco matrix in all observed species is associated with thylakoid membranes (Meyer et al. 2017), which are thought to deliver CO_2_ to Rubisco (Fig. 2) (Hennacy and Jonikas 2020). The simplest pyrenoids consist of a Rubisco matrix either embedded between thylakoid membranes, such as that of the coccolithophore *Emiliania huxleyi* (Buma et al. 2000), or projecting out of the chloroplast into the cytoplasm, such as that of the diatom *Attheya ussurensis* (Bedoshvili et al. 2009) (Fig. 3). In most species, the thylakoid membranes traverse the pyrenoid matrix either as sheets, as in the euglenophyte *Euglena carterae* (Kusel-Fetzmann and Weidinger 2008), or tube-like structures, as in the chlorophyte *Heveochlorella hainangensis* (Zhang et al. 2008). In some species, Rubisco matrices lack traversing thylakoids but are surrounded by polysaccharide deposits that potentially act as CO_2_ leakage barriers, as in the chlorophyte *Micromonas pusilla* (van Baren et al. 2016). The most elaborate pyrenoid morphologies consist of all three sub-structures: thylakoids traversing a Rubisco matrix that is encased in a starch sheath, as found in Chlamydomonas (Fig. 1B). In this section, we describe what is known about each of the pyrenoid sub-compartments in Chlamydomonas and give a brief introduction to what is known about these structures in other species.

### The CO_2_-fixing pyrenoid matrix is a phase-separated condensate of Rubisco and a linker protein

Rubisco makes up approximately 90% of the protein content of the pyrenoid matrix (Holdsworth 1971). Based on transmission electron microscopy (TEM) images, the matrix appears crystalline in several species (Holdsworth 1968; Kowallik 1969; Bertagnolli and Nadakavukaren 1970) and amorphous in others (Griffiths 1970; Meyer et al. 2012).

The Chlamydomonas pyrenoid matrix was recently shown to be a liquid-like phase-separated condensate (Freeman Rosenzweig et al. 2017). Fluorescence recovery after photobleaching experiments indicated that the matrix mixes internally on a timescale of approximately 20 seconds (Freeman Rosenzweig et al. 2017), similar to that observed for other liquid-like compartments such as P granules and nucleoli (Bracha et al. 2019). Furthermore, the Rubisco matrix exhibits other liquid-like behaviors, including division by fission, and dissolution into the chloroplast during cell division and under high CO_2_ conditions (>0.40% CO_2_). Rubisco in the Chlamydomonas pyrenoid matrix was found by cryo-electron tomography (cryo-ET) to lack the long-range order characteristic of a crystal; instead, the distribution of Rubisco fits well with a simple model for the distribution of particles in a liquid (Freeman Rosenzweig et al. 2017). This description of the pyrenoid matrix as a phase-separated condensate likely applies to pyrenoids in other species, as it explains observations such as the spheroidal shape of most pyrenoids (Fig. 3), the rapid appearance and disappearance of pyrenoids during cell division (Brown et al. 1967; Retallack and Butler 1970), and their division by fission (Brown and Bold 1964; Brown et al. 1967).

For decades, only two matrix proteins were known: Rubisco and Rubisco activase (RCA1, Cre04.g229300) (Holdsworth 1971; Vladimirova et al. 1982; McKay and Gibbs 1991), and the mechanism by which Rubisco is densely clustered in the pyrenoid matrix was a mystery. In 2016, the repeat protein EPYC1 was proposed to link individual Rubiscos to form the matrix in Chlamydomonas (Mackinder et al. 2016). EPYC1 localizes to the pyrenoid matrix and is one of the most abundant proteins in the pyrenoid after Rubisco (Mackinder et al. 2016; Hammel et al. 2018). In *epyc1* mutant cells, the majority of Rubisco is dispersed in the chloroplast outside of the pyrenoid, indicating that EPYC1 plays a major role in Rubisco localization to the matrix (Mackinder et al. 2016). Purified Rubisco and EPYC1 can phase-separate with each other to form liquid-like droplets in vitro (Wunder et al. 2018), suggesting that these two proteins are sufficient for driving the formation of the liquid-like pyrenoid matrix.

**Figure 4.**
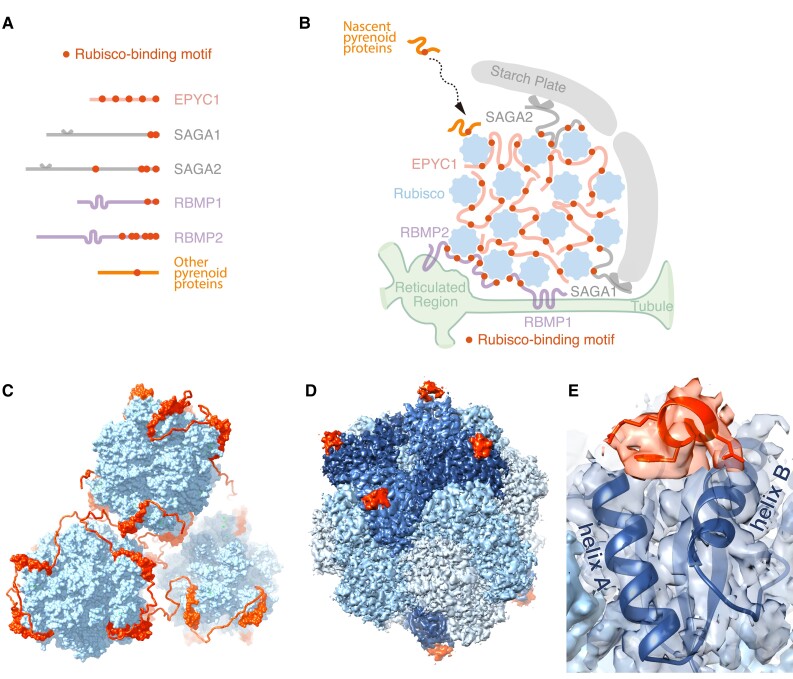
A common Rubisco-binding motif mediates the assembly of the major compartments of the pyrenoid. **A)** In Chlamydomonas, many pyrenoid-localized proteins contain at least one Rubisco-binding motif. **B)** The Rubisco-binding motif mediates the assembly of the three pyrenoid sub-compartments. The motifs on EPYC1 link Rubisco to form the pyrenoid matrix (He et al. 2020). The motifs on the tubule-localized transmembrane proteins RBMP1 and RBMP2 are proposed to connect the Rubisco to the tubules, and the motifs on the putative starch-binding proteins SAGA1 and SAGA2 are proposed to mediate interactions between the matrix and the surrounding starch sheath. A Rubisco-binding motif was also shown to be necessary and sufficient to target a nascent protein to the pyrenoid (Meyer et al. 2020b). **C)** A model illustrating how EPYC1 (red) clusters Rubisco (blue) in the pyrenoid matrix. **D)** The Rubisco-binding motif of EPYC1 (red) binds to the Rubisco small subunit (dark blue) (He et al. 2020); other Rubisco-binding motifs in Chlamydomonas are expected to bind to the same site. **E)** The motif binds between two alpha-helices of the Rubisco small subunit.

Rubisco is an oligomeric holoenzyme with eight identical large subunits and eight identical small subunits. A structural study using cryo-electron microscopy (cryo-EM) found that EPYC1 directly binds to Rubisco on the two alpha-helices of each Rubisco small subunit through salt bridges and hydrophobic interactions (Fig. 4) (He et al. 2020). This structure is consistent with previous genetic studies showing that these alpha helices are important for the formation of the pyrenoid and for the Rubisco–EPYC1 interaction (Meyer et al. 2012; Atkinson et al. 2019). Each Rubisco holoenzyme has eight EPYC1-binding sites and each EPYC1 has five Rubisco-binding sites (Fig. 4, C to E), allowing the two proteins to form an interdependent network that clusters Rubisco together in the pyrenoid matrix. The low binding affinity (approximately 3 mM) between individual EPYC1–Rubisco binding site is consistent with the principle that biopolymer phase separation is mediated by weak multivalent interactions (Li et al. 2012).

Beyond Rubisco, RCA1, and EPYC1, ten additional proteins show exclusive localization or enrichment in the Chlamydomonas pyrenoid matrix when examined as fluorescently tagged proteins (Fig. 1; Table 1). These proteins are the putative S-adenosyl-L-methionine-dependent methyltransferase SMM7 (Cre03.g151650) (Mackinder et al. 2017), the predicted xylulose-1,5-bisphosphate (XuBP) phosphatase conserved in the Plantae and diatoms 2 (CPLD2, Cre03.g206550), the putative histone deacetylase HDA5 (Cre06.g290400), uncharacterized proteins encoded by Cre16.g648400, Cre13.g573250, Cre16.g663150, Cre02.g143635 (Wang et al. 2022), thiosulfate sulfurtransferase16 (STR16, Cre13.g573250), STR18 (Cre16.g663150), and ATP-binding cassette F like-protein 6 (ABCF6, Cre06.g271850) (Lau et al. 2023) (Table 1). CPLD2 is the homolog of the highly selective Arabidopsis XuBP phosphatase AtCbbY (At3g48420), which converts the Rubisco inhibitor XuBP to a non-inhibitory compound that can be recycled back to the Rubisco substrate RuBP (Bracher et al. 2015). The pyrenoid localization of CPLD2 suggests that it may also convert XuBP to RuBP in the pyrenoid. Both HDA5 and the protein encoded by Cre16.g648400 have predicted “Rubisco-binding motifs” (Fig. 4) (discussed in a later section) (Meyer et al. 2020b), suggesting that they bind Rubisco in the pyrenoid matrix (Wang et al. 2022). STR16, STR18, and the proteins encoded by Cre13.g573250 and Cre16.g663150 are all predicted to be thiosulfate sulfurtransferases. In addition, STR16 and STR18 contain a rhodanese domain, which is predicted to function in disulfide bond formation and iron-sulfur cluster biosynthesis (Lau et al. 2023). ABCF6 is a predicted member of the ATP-binding cassette F (ABCF) protein family that regulates translation via binding to ribosomes (Lau et al. 2023).

**Table 1.** Summary of Chlamydomonas pyrenoid–specific proteins whose localization has been confirmed.

Protein name	Gene ID	Subpyrenoid localization		Localization reference	Reported or predicted functions
ABCF6	Cre06.g271850	Matrix	①	Lau et al. (2023)	Predicted ATP-binding cassette family F like protein
CPLD2	Cre03.g206550	Matrix	①	Wang et al. (2022)	Homolog of the Arabidopsis XuBP phosphatase CbbY (At3g48420)
EPYC1/ LCI5	Cre10.g436550	Matrix	①	Mackinder et al. (2016)	Rubisco linker, phase-separates with Rubisco to form the pyrenoid matrix
HDA5	Cre06.g290400	Matrix	①	Wang et al. (2022)	Predicted histone deacetylase
rbcL	CreCp.g802313	Matrix	①	Holdsworth (1971); Mackinder et al. (2017)	Large and small subunits of the Rubisco holoenzyme, which fixes CO_2_ and produces 3-PG and 2-PG
RBCS1	Cre02.g120100				
RBCS2	Cre02.g120150				
RCA1	Cre04.g229300	Matrix	①	Vladimirova et al. (1982); Lacoste-Royal (1987); McKay et al. (1991); Mackinder et al. (2017)	Rubisco activase
STR16	Cre13.g573250	Matrix	①	Wang et al. (2022); Lau et al. (2023)	Predicted thiosulfate sulfurtransferase containing a rhodanese domain
STR18	Cre16.g663150	Matrix	①	Wang et al. (2022); Lau et al. (2023)	Predicted thiosulfate sulfurtransferase containing a rhodanese domain
–	Cre16.g648400	Matrix	①	Wang et al. (2022)	–
–	Cre02.g143635	Matrix	①	Wang et al. (2022)	–
CAH3	Cre09.g415700	Tubules	②	Sinetova et al. (2012)	Alpha-type carbonic anhydrase
CAS1	Cre12.g497300	Tubules	②	Wang et al. (2016)	Calcium-mediated regulator of the expression of some CCM-related genes
CYN7	Cre12.g544150	Tubules	②	Wang et al. (2022)	Predicted peptidyl-prolyl cis-trans isomerase
CYN20-6	Cre12.g544114	Tubules	②	Wang et al. (2022)	Predicted peptidyl-prolyl cis-trans isomerase
DEG8	Cre01.g028350	Tubules	②	Wang et al. (2022)	Predicted DegP-type protease, Arabidopsis homolog is involved in the degradation of photodamaged PSII reaction center protein D1
HCF136	Cre06. g273700	Tubules	②	Wang et al. (2022)	PS II stability/assembly factor HCF136
PSAH	Cre07. g330250	Tubules	②	Mackinder et al. (2017)	PSI subunit
PSBP1	Cre12.g550850	Tubules	②	Wang et al. (2022)	Predicted oxygen-evolving enhancer protein 2 of PS II
RBMP1	Cre06. g261750	Tubules	②	Meyer et al. (2020b)	Proposed to mediate pyrenoid matrix connection to tubules
TEF14	Cre06.g256250	Tubules	②	Wang et al. (2022)	Predicted thylakoid-luminal protein, no labeled domains
-	Cre03.g172700	Tubules	②	Lau et al. (2023)	Protein with multiple predicted Rubisco-binding motifs
CGLD14	Cre10.g446350	Pyrenoid center (reticulated region)	⑤	Wang et al. (2022)	PSBP domain-containing protein 3, conserved in the green lineage and diatoms
PNU1	Cre03.g183550	Pyrenoid center (reticulated region)	⑤	Wang et al. (2022)	PROTEIN F23H11.5, has a bifunctional nuclease domain
RBMP2	Cre09.g416850	Tubules (reticulated region)	⑤	Meyer et al. (2020b)	Proposed to mediate pyrenoid matrix connection to tubules
PSBP4	Cre08.g362900	Thylakoid lumen puncta	⑨	Mackinder et al. (2017)	Luminal PsbP-like protein, Arabidopsis homolog is essential for PS I assembly and function
SAGA1	Cre11.g467712	Puncta surrounding the matrix	④	Itakura et al. (2019); Meyer et al. (2020b)	Proposed to mediate adherence of the starch sheath to the matrix
SAGA2	Cre09.g394621	Interface between matrix and starch sheath	④	Meyer et al. (2020b)	Proposed to mediate adherence of the starch sheath to the matrix
SMC7	Cre17.g720450	Puncta surrounding the matrix	④	Lau et al. (2023)	Proposed to have a similar function as SAGA1
SBE3	Cre10.g444700	Starch sheath	③	Mackinder et al. (2017)	Conserved starch-branching enzyme
STA2	Cre17.g721500	Starch sheath	③	Mackinder et al. (2017)	Granule-bound starch synthase involved in amylose biosynthesis and the biosynthesis of long chains in amylopectin
–	Cre09.g394547	Starch sheath	③	Wang et al. (2022)	Predicted cyclomaltodextrin glucanotransferase/cyclodextrin glycosyltransferase
–	Cre09.g415600	Starch sheath	③	Wang et al. (2022)	Predicted glucan 1,4-alpha-glucosidase/Lysosomal alpha-glucosidase; has a starch-binding domain
LCI9	Cre09.g394473	Gaps between starch plates	⑦	Mackinder et al. (2017)	Contains 2 starch-binding domains; may help ensure a close fit for adjacent starch plates
LCIB	Cre10.g452800	Gaps between starch plates and tubules (very low CO_2_)	⑧	Yamano et al. (2010); Wang and Spalding (2014b); Mackinder et al. (2017)	Beta-type carbonic anhydrase
LCIC	Cre06.g307500	Gaps between starch plates and tubules (very low CO_2_)	⑧	Yamano et al. (2010); Mackinder et al. (2017)	Beta-type carbonic anhydrase
–	Cre09.g394510	Starch-matrix interface and gaps between starch plates	④⑦⑧	Lau et al. (2023)	Contains a CBM20 starch-binding domain and a t-SNARE domain; proposed to be involved in membrane remodeling of the pyrenoid tubules; could be involved in membrane remodeling of the pyrenoid tubules
MDH1	Cre03.g194850	Pyrenoid periphery	–	Wang et al. (2022)	Malate dehydrogenase
MIND1	Cre12.g522950	Pyrenoid periphery	–	Wang et al. (2022)	Homolog of the Arabidopsis chloroplast division site regulator MinD1

The circled numbers indicating the subpyrenoid localizations are coordinated with the circled numbers in Fig. 1C.

### Pyrenoid-associated membranes likely supply Rubisco with concentrated CO_2_

The Rubisco matrix in all known pyrenoids is in contact with a portion of the thylakoid membranes of the chloroplast (Meyer et al. 2017), consistent with the idea that these membranes perform the essential function of supplying CO_2_ to Rubisco (Pronina and Semenenko 1990; Raven 2008). A broad range of morphologies has been observed in different species for these pyrenoid-associated thylakoids (Fig. 3). Some species have a single traversing membrane, some have multiple parallel or interconnected membranes, and some have more complex morphologies, such as the undulating membranes found in species of the red algal genus *Porphyridium* (Nelson and Ryan 1988) (Fig. 3). Some pyrenoids, such as in the dinoflagellate *Podolampas bipes* (Schnepf and ElbräChter 1999) (Fig. 3), have no observed traversing membranes and instead are embedded between thylakoid membranes in the chloroplast. Given that CO_2_ diffuses rapidly relative to the rate of its fixation by Rubisco, the exact location of CO_2_ release within the pyrenoid likely has little effect on the distribution of CO_2_ within the pyrenoid; thus, a broad range of membrane morphologies can effectively supply CO_2_ to Rubisco (Fei et al. 2022).

In Chlamydomonas, the thylakoid membranes extend into the Rubisco matrix to form pyrenoid tubules whose lumina are continuous with the thylakoid lumen (Fig. 1, C and D) (Sager and Palade 1954, 1957; Ohad et al. 1967a). Traditional 2D TEM images have shown that thylakoid sheets near the pyrenoid are directed toward the gaps of the starch sheath (Sager and Palade 1954, 1957; Ohad et al. 1967a). This observation was corroborated by cryo-ET, which showed in 3D that thylakoid sheets merge with each other as they pass through gaps in the starch sheath to form cylindrical pyrenoid tubules that traverse the Rubisco matrix (Fig. 1, C and D) (Engel et al. 2015). In the center of the pyrenoid, the tubules converge to form a complex interconnected network known as the reticulated region (Fig. 1, C and D) (Engel et al. 2015; Meyer et al. 2020b).

Inside each Chlamydomonas tubule, there are two to eight smaller tubes called minitubules (Ohad et al. 1967b; Engel et al. 2015) (Fig. 1, C and D). Minitubules appear to provide conduits from the inter-thylakoid stromal space to the pyrenoid matrix (Engel et al. 2015). These minitubules have been proposed to facilitate the diffusion of small molecules such as RuBP and 3-PG between these two compartments (Engel et al. 2015; Küken et al. 2018); however, their internal diameters of approximately 3.5 ± 0.5 nm are likely too small to mediate a substantial flux of metabolites. Curiously, minitubules have not been observed in any species other than Chlamydomonas (Meyer et al. 2017), although this could be due to limitations in TEM imaging. The function of minitubules remains unknown, and the mechanisms by which the intricate morphology of the different regions of the pyrenoid-traversing membranes is achieved is unknown in any organism.

It is notable that Chlamydomonas mutants that lack a Rubisco matrix still have tubule networks in the canonical location within the chloroplast (Caspari et al. 2017; He et al. 2020), indicating that the tubules can form in the absence of the matrix and suggesting that the location of the pyrenoid could be determined by the tubules. However, the molecular basis for the localization of the tubules remains unknown.

The delivery of concentrated CO_2_ to Rubisco in the matrix is thought to be mediated by carbonic anhydrases that convert HCO_3_^−^ into CO_2_ in the lumen of the pyrenoid-traversing membranes (Pronina and Semenenko 1990; Raven 2008). In Chlamydomonas, the carbonic anhydrase that mediates this key step is CAH3 (Karlsson et al. 1998; Hanson et al. 2003). Consistent with this role, Chlamydomonas mutants lacking functional CAH3 have a severe growth defect when grown in limiting CO_2_ conditions (Spalding et al. 1983; Funke et al. 1997; Karlsson et al. 1998) and over-accumulate HCO_3_^−^ within the mutant cells (Spalding et al. 1983). CAH3 localizes to the thylakoid lumen (Karlsson et al. 1998) and becomes enriched in the pyrenoid tubules during activation of the CCM in transitions from high CO_2_ to limiting CO_2_ (Blanco-Rivero et al. 2012; Sinetova et al. 2012) and from dark to light (Mitchell et al. 2014). How CAH3 relocalizes to the tubules remains unknown.

Recent studies in Chlamydomonas have identified additional pyrenoid tubule–localized proteins that perform various functions. Like CAH3, the Ca^2+^-binding protein calcium sensing receptor (CAS, Cre12.g497300) relocalizes to the pyrenoid tubules upon CCM induction (Wang et al. 2016; Yamano et al. 2018). CAS is a putative Rhodanese-like Ca^2+^-sensing receptor that regulates the expression of several CCM-related genes, including *HLA3* and *LCIA* (Wang et al. 2016). Upon activation of the CCM, CAS switches from being dispersed across the chloroplast to being associated with the pyrenoid tubules (Wang et al. 2016). This change is accompanied by an increase in Ca^2+^ in the pyrenoid (Wang et al. 2016). The role of Ca^2+^ in the pyrenoid, the mechanism of CAS relocalization, and the purpose of CAS signaling in the CCM remain unclear.

Two other tubule-localized proteins are Rubisco-binding membrane protein 1 (RBMP1, encoded by Cre06.g261750) and RBMP2 (Cre09.g416850) (Fig. 4). Both proteins bind to Rubisco in vitro (Meyer et al. 2020b) and in vivo (Mackinder et al. 2017), suggesting that they may promote interactions between the Rubisco matrix and the pyrenoid tubules. RBMP1 is associated with peripheral tubular regions, whereas RBMP2 localizes to the central reticulated region of the tubules (Meyer et al. 2020b). Intriguingly, RBMP2 contains a rhodanese domain, as do STR16, STR18, and CAS, but the function of these rhodanese domains in these proteins remains to be determined. The putative roles of RBMP1 and RBMP2 in linking matrix to tubules also remain to be tested.

In addition to RBMP2, the proteins pyrenoid nuclease 1 (PNU1, Cre03.g183550) and conserved in the green lineage and diatoms 14 (CGLD14, Cre10.g446350) appear to localize to the reticulated region of the tubules (Wang et al. 2022). PNU1 is a bifunctional nuclease domain-containing protein. As oxidized RNA was also localized to the pyrenoid in Chlamydomonas (Zhan et al. 2015), the pyrenoid localization of PNU1 suggests that the pyrenoid might be a site of oxidized RNA degradation (Wang et al. 2022). CGLD14 is conserved in the green lineage and diatoms and is also named PSBP-domain-containing protein 3 (PPD3).

Multiple components of the electron transport chain are present in the Chlamydomonas pyrenoid tubules, including subunits of photosystem I (PSI) (Photosystem I reaction center subunit V [PSAG, Cre12.g560950], Photosystem I reaction center subunit H [PSAH, Cre07.g330250], Photosystem I reaction center subunit K [PSAK, Cre17.g724300], and chloroplast-localized ferredoxin [FDX1, Cre14.g626700]), photosystem II (PSII) (PsbP-like protein 3 [PSBP3, Cre12.g509050], PSBP4 [Cre08.g362900], Photosystem II oxygen evolution enhancer protein 3 [PSBQ, Cre08.g372450], and Photosystem II subunit R [PSBR, Cre06.g261000]), cytochrome *b*_6_*f* (CYC6, Cre16.g651050), and ATP synthase (ATPC, Cre06.g259900) (Mackinder et al. 2017). However, despite some components of the O_2_-evolving PSII being present in Chlamydomonas pyrenoid-traversing membranes, other PSII components (such as subunit 1 of the PSII oxygen-evolving enhancer protein 1 [OEE1 or PSBO1, Cre09.g396213] and PSII intrinsic core polypeptides D2 [psbD, CreCp.g802329] and P5 [PSBP5, Cre09.g389578]) appear to be absent based on immunogold labeling (de Vitry et al. 1989; McKay and Gibbs 1991). Relatedly, PSII was found to be inactive in the pyrenoid of the red alga *Porphyridium cruentum* based on cytochemical assays in which PSII activity was detected through the production of osmiophilic diformazan upon the photoreduction of tetrazolium salts (McKay and Gibbs 1990, 1991). Minimizing PSII activity in pyrenoid-traversing thylakoids may be a strategy for minimizing O_2_ levels within the pyrenoid, which may help maintain a high CO_2_ to O_2_ ratio around Rubisco (McKay and Gibbs 1991). The electron transport chain components found in the pyrenoid may therefore be in assembly intermediates, in inactive complexes undergoing repair, or may have different functions from those found in stromal thylakoids. This hypothesis is supported by the pyrenoid localization of PSBP4, which is a homolog of the Arabidopsis PSII repair protein PSBP-LIKE PROTEIN1 (PPL1, encoded by At3g55330) and interacts with four known PSI assembly factors (Mackinder et al. 2017).

Eight other proteins have recently been localized to the pyrenoid tubules in Chlamydomonas: Deg protease 8 (DEG8, Cre01.g028350), the cyclophilins CYN7 (Cre12.g544150) and CYN20-6 (Cre12.g544114), Photosystem II subunit P1 (PSBP1, Cre12.g550850), the PSII stability/assembly factor high chlorophyll fluorescence 136 (HCF136, Cre06.g273700), the thylakoid luminal protein thylakoid luminal factor 14 (TEF14, Cre06.g256250), uncharacterized proteins encoded by Cre03.g198850 (Wang et al. 2022), and Cre03.g172700 (Lau et al. 2023) (Table 1). DEG8 is a predicted DegP-type protease, while CYN7 and CYN20-6 are two predicted peptidyl-prolyl cis-trans isomerases. The pyrenoid tubules may thus be involved in protein folding, degradation, and/or import of new proteins into the pyrenoid (Wang et al. 2022). The protein encoded by Cre03.g172700 is predicted to contain a long central alpha-helix and four “Rubisco-binding motifs” (discussed in a later section), which might allow it to act as a potential pyrenoid tether between the pyrenoid matrix and tubules alongside RBMP1 and RBMP2 (Lau et al. 2023).

### A polysaccharide sheath likely serves as a CO_2_ diffusion barrier

Polysaccharide deposits are associated with the pyrenoids of some species in every major algal lineage except the diatoms and coccolithophores (Fig. 3). In red algae and green algae, the polysaccharide that makes up these deposits is starch, whereas different polymers are used in other lineages, such as paramylon in the case of euglenoid algae (Nudelman et al. 2006; Suzuki and Suzuki 2013; Ball et al. 2015). Green algae produce starch in the chloroplast, whereas all other lineages produce their polysaccharide deposits in the cytosol (with the exception of the cryptophytes, which produce starch in the periplastid) (Suzuki and Suzuki 2013; Ball et al. 2015). Presumably because of these differences, species from all lineages except the green algae only have polysaccharides associated with their pyrenoids if they have a stalked or bulging pyrenoid that projects into the cytoplasm (Meyer et al. 2017) (Fig. 3). In these cases, the polysaccharide structures are separated from the Rubisco matrix by the chloroplast envelope (Meyer et al. 2017). The association of polysaccharide deposits with pyrenoids even when separated by membranes further implicates these structures in pyrenoid function. In Chlamydomonas, the starch sheath is a shell-like structure made by curved starch granules around the pyrenoid matrix (Fig. 1). Small gaps between starch plates allow the tubules to penetrate through into the matrix.

Available evidence suggests that the pyrenoid polysaccharide sheath, when present, serves as a barrier to slow the escape of CO_2_ from the pyrenoid, allowing a higher concentration of CO_2_ to be maintained in the pyrenoid and decreasing the energetic costs of CO_2_ concentration (Toyokawa et al. 2020; Fei et al. 2022). The most convincing evidence to date supporting this function comes from the decreased CCM efficacy observed under very low CO_2_ in the Chlamydomonas *sta2-1* mutant (defective in starch synthase 2 [STA2, encoded by Cre17.g721500]), which has a thinner starch sheath but otherwise apparently normal localization of key proteins (Toyokawa et al. 2020).

The pyrenoid starch sheath granules in Chlamydomonas are different from the stromal starch granules in their shape, composition, and the conditions under which they accumulate. The granules that make up the starch sheath are more curved than stromal granules, which are globular in shape. The molecular composition of starch consists of alternating amorphous layers of amylose and crystalline layers of amylopectin (Zeeman et al. 2010). Compared with stromal starch, pyrenoidal starch has less amylose but more amylopectin content (Libessart et al. 1995; Findinier et al. 2019). Both amylose and amylopectin have been shown to decrease O_2_ gas permeability in vitro (Forssell et al. 2002), which further supports the possible function of the starch sheath in slowing down the escape of leaking CO_2_ from the matrix. Relatedly, the molecular structure of starch varies depending on the algal lineage (Suzuki and Suzuki 2013; Ball et al. 2015), which could have implications for the ability of starch to prevent CO_2_ diffusion in different species. In addition to differences in their shape and composition, pyrenoid starch sheath granules and stromal starch granules also accumulate under different conditions. When Chlamydomonas cells are grown in unfavorable conditions such as during nitrogen starvation, stromal starch content increases while that of pyrenoid starch decreases, as starch metabolism rapidly switches from pyrenoidal to storage biosynthesis (Kuchitsu et al. 1988; Findinier et al. 2019). However, when cells are moved from high CO_2_ (4%) to low CO_2_ (air-level), pyrenoid starch accumulates rapidly within hours and stromal starch is degraded (Kuchitsu et al. 1988).

Several proteins have been implicated in the formation and degradation of the starch sheath in Chlamydomonas. The protein StArch Granules Abnormal 1 (SAGA1, encoded by Cre11.g467712), which contains a putative starch-binding domain, localizes to distinct puncta at the pyrenoid matrix/starch interface (Figs. 1, C and D and 4B) (Itakura et al. 2019; Meyer et al. 2020b). Abnormally elongated and thinner starch granules were observed in *saga1* mutant cells, indicating that SAGA1 is required for normal starch sheath formation (Itakura et al. 2019). A recent study suggested that SAGA1 is also necessary for relocalizing CAS and LCIB to the pyrenoid under limiting CO_2_ conditions and for CAS-dependent retrograde signaling regulation of nuclear genes encoding CO_2_ and HCO_3_^−^ transporters (Shimamura et al. 2023). Another protein, bimodal starch granule 1 (BSG1, Cre02.g091750), may be involved in the degradation of the starch sheath during the transition from low CO_2_ to high CO_2_ (Findinier et al. 2019).

High-throughput studies have identified other proteins that could potentially be involved in the formation of the pyrenoid starch sheath (Table 1) (Mackinder et al. 2017; Meyer et al. 2020b). SAGA2 (Cre09.g394621) is a protein that shares 30% sequence identity with SAGA1 and also has a predicted starch-binding domain (Meyer et al. 2020b). Like SAGA1, SAGA2 also localizes to the pyrenoid matrix/starch interface (Fig. 4B), although its function is currently unknown. Two other proteins, granule-bound STA2 (Delrue et al. 1992; Maddelein et al. 1994) and starch-branching enzyme 3 (SBE3, Cre10.g444700), localize around the pyrenoid periphery, forming a plate-like pattern (Mackinder et al. 2017), which suggests that they may contribute to the biosynthesis of the starch sheath (Table 1). LCI9 (Cre09.g394473), which contains two starch-binding domains and is predicted to function as a glucan 1,4-α-glucosidase, localizes in a mesh structure between the gaps of the starch sheath, suggesting that it may degrade starch at the gaps between starch plates to ensure a close fit between adjacent plates (Mackinder et al. 2017). Further studies of these starch-associated proteins are needed to understand their functions in the biogenesis of the Chlamydomonas starch sheath.

Six new pyrenoid-periphery proteins were recently identified in Chlamydomonas: MIND1 (Cre12.g522950), malate dehydrogenase 1 (MDH1, Cre03.g194850), uncharacterized proteins encoded by Cre09.g394547, Cre09.g415600 (Wang et al. 2022), structural maintenance of chromosomes 7 (SMC7, Cre17.g720450), and uncharacterized protein encoded by Cre09.g394510 (Lau et al. 2023) (Table 1). The location of most of these newly identified proteins relative to the starch sheath remains unclear. Interestingly, MIND1 is a homolog of the Arabidopsis chloroplast division site regulator MinD1 (At5g24020), suggesting that MIND1 could potentially play a role in coordinating pyrenoid fission or dissolution with chloroplast division in Chlamydomonas (Colletti et al. 2000; Freeman Rosenzweig et al. 2017; Wang et al. 2022). SMC7 shows a punctate localization similar to that of SAGA1 and is annotated as a member of the SMC family (Lau et al. 2023). SAGA1 and SAGA2 are also annotated as members of this family, which suggests that SMC7 might function similarly to SAGA1 and SAGA2. The protein encoded by Cre09.g394510 contains a starch-binding domain and localizes to the starch-matrix interface and the gaps between starch plates. It contains a predicted t-SNARE domain, which mediates vesicle fusion, suggesting that it may be involved in membrane remodeling of the pyrenoid tubules (Lau et al. 2023).

### A Rubisco-binding motif mediates pyrenoid assembly

As previously discussed, the repeat protein EPYC1 has five Rubisco-binding regions critical for pyrenoid matrix assembly in Chlamydomonas (He et al. 2020). Intriguingly, similar sequences to the EPYC1 Rubisco-binding region have been identified on many other pyrenoid-localized proteins (Fig. 4, A and B) (Meyer et al. 2020b). These sequences, including the Rubisco-binding region on EPYC1, have been named “Rubisco-binding motifs.” The motifs on other pyrenoid proteins show similar binding affinity to Rubisco as the motifs on EPYC1 (whose K_D_ is approximately 3 mM) (He et al. 2020; Meyer et al. 2020b). Due to sequence similarity, the motifs on other proteins are believed to bind to the same alpha-helices of Rubisco small subunits as EPYC1 (Fig. 4, C–E).

The Rubisco-binding motif was shown to be necessary and sufficient for targeting a protein to the pyrenoid matrix (Meyer et al. 2020b). These observations suggest that nascent pyrenoid proteins with copies of the motif diffuse around the chloroplast stroma until they encounter the matrix, where they are captured by binding to Rubisco (Fig. 4B). One open question is how proteins are targeted to the matrix when a full starch sheath has been assembled because stromal proteins would then not have direct access to Rubisco.

In addition to targeting proteins to the matrix, the Rubisco-binding motif has been proposed to anchor the Rubisco matrix to the pyrenoid tubules and connect the starch sheath to the matrix (Meyer et al. 2020b). The Rubisco-binding motif-containing proteins RBMP1 and RBMP2 localize to the tubules, suggesting that they target a layer of Rubisco to the tubules. From there, EPYC1 may be able to connect additional Rubiscos, causing the matrix to condense around the entire tubule network. The proteins SAGA1 and SAGA2 also contain Rubisco-binding motifs in addition to their starch-binding domains, which suggests that they might link the matrix to the starch sheath (Fig. 4B) (Meyer et al. 2020b).

The identification of the Rubisco-binding motif and the hypothesis that proteins with this motif link the three pyrenoid sub-compartments together in Chlamydomonas explains the initially puzzling difference between the phenotypes of a mutant lacking functional EPYC1 and mutants with disrupted EPYC1-binding sites on Rubisco small subunits (He et al. 2020; Meyer et al. 2020b). In a mutant lacking EPYC1, a minimal pyrenoid can still be observed (Mackinder et al. 2016); however, mutants with disrupted EPYC1-binding sites on Rubisco small subunits lack a pyrenoid altogether (He et al. 2020; Meyer et al. 2012, 2020b). These findings can be reconciled when considering that, in the mutant lacking EPYC1, proteins other than EPYC1 that have the Rubisco-binding motif (potentially including RBMP1 and RBMP2) can still bind to Rubisco and form the observed much smaller pyrenoid-like structure that still contains tubules and a starch sheath but lacks a canonical matrix.

The sequences of the Rubisco-binding motifs and their binding sites on Rubisco are conserved in the order Volvocales to which Chlamydomonas belongs, but the motif has not been found in any other algal lineages (Meyer et al. 2020b). Assuming that pyrenoids convergently evolved, it is possible that the assembly of pyrenoids via common Rubisco-binding motifs may broadly apply to pyrenoids across the algal tree of life, although the specific sequences may differ in different algal lineages (Meyer et al. 2020b).

### Other candidate pyrenoid components in Chlamydomonas

Physical interactors of proteins that localize to the pyrenoid were identified using large-scale affinity-purification mass spectrometry (Mackinder et al. 2017). Using this method, 513 interactions involving 398 proteins were identified (Mackinder et al. 2017).

In a parallel study, Chlamydomonas pyrenoids were purified and their proteome was analyzed, identifying 190 proteins in total (Zhan et al. 2018). Of the 190 candidate pyrenoid proteins identified, many have confirmed or predicted functions that are known or proposed to occur in pyrenoids, such as the CCM, starch metabolism, or RNA metabolism and translation. Additional proteins suggestive of new pyrenoid functions in tetrapyrrole and chlorophyll synthesis, carotenoid metabolism, or amino acid metabolism were also identified. Future work on these uncharacterized candidate pyrenoid proteins will yield a better understanding of the biogenesis, function, and regulation of the pyrenoid.

## Dynamics and regulation

Pyrenoids in various species are dynamic, showing noticeable morphological changes under different growth conditions and during cell division (Brown and Bold 1964; Brown et al. 1967; Goodenough 1970; Retallack and Butler 1970). The newly reported liquid-like nature of the Chlamydomonas pyrenoid (Freeman Rosenzweig et al. 2017) provides a new framework for thinking about the biophysics underlying pyrenoid dissolution, condensation, and division by fission. Pyrenoid dynamics are likely highly regulated, but the underlying regulatory mechanisms remain to be discovered.

### The pyrenoid forms in response to limiting CO_2_ levels under constant light

When Chlamydomonas cells are transferred from high CO_2_ to limiting CO_2_ under constant light conditions, the pyrenoid matrix grows within one hour (Kuchitsu et al. 1991; Ramazanov et al. 1994), presumably by relocalization of Rubisco from the chloroplast stroma to the pyrenoid matrix. The starch sheath starts to form within one hour after transfer from high to low CO_2_ as well and is fully formed after about five hours (Kuchitsu et al. 1988; Ramazanov et al. 1994).

The expression of many CCM-related genes, including those encoding confirmed pyrenoid proteins such as EPYC1, STA2, and CAH3, is upregulated during the transition from high to low CO_2_ (Brueggeman et al. 2012; Fang et al. 2012), which is consistent with the expansion of the pyrenoid matrix and the formation of the starch sheath. The Chlamydomonas CCM “master regulator” inorganic carbon (Ci) acquisition 5 (CIA5, Cre02.g096300, also known as CCM1) is required for the transcriptional upregulation of *CAH3*, *EPYC1*, *STA2*, *LCIB*, *LCIC*, and *SMM7* in response to the transition from high CO_2_ to low CO_2_ (Fang et al. 2012; Santhanagopalan et al. 2021), although CIA5 may also have other non-CCM-related functions (Moroney et al. 1989; Marek and Spalding 1991; Miura et al. 2004; Wang et al. 2005; Fang et al. 2012; Redekop et al. 2022). CIA5 has zinc-binding activity and was proposed to be a transcription factor (Fukuzawa et al. 2001; Xiang et al. 2001; Kohinata et al. 2008), but this has not been confirmed because no DNA-CIA5 complexes have been identified. Additionally, the *CIA5* regulatory mechanism is not well understood. *CIA5* transcript and CIA5 protein levels are similar in high–CO_2_-grown and low–CO_2_-grown unsynchronized wild-type cells (Wang et al. 2005; Fang et al. 2012); thus, CIA5 activity may be regulated by posttranslational modifications (Fukuzawa et al. 2001; Xiang et al. 2001; Wang et al. 2005; Brueggeman et al. 2012; Chen 2016). CIA5 regulates the transcription factor low-CO_2_ stress response 1 (LCR1, Cre09.g399552), which is known to directly regulate the expression of *CAH1* (Cre04.g223100), *LCI1* (Cre03.g162800), and *LCI6* (Cre12.g553350) (Yoshioka et al. 2004).

Posttranslational modifications are thought to regulate the functions of several essential pyrenoid proteins. The Rubisco linker EPYC1/LCI5 was reported to be phosphorylated under low CO_2_ conditions but not under high CO_2_ (Turkina et al. 2006), although the functional implications of this phosphorylation are unknown. The relocalization of CAH3 from the stromal thylakoids to the pyrenoid tubules under low CO_2_ conditions as well as the functions of HLA3 and LCIC under low CO_2_ and very low CO_2_ have also been proposed to be regulated by phosphorylation (Blanco-Rivero et al. 2012; Wang et al. 2014). LCIB is glutathionylated during acclimation to limiting CO_2_ (Zaffagnini et al. 2012). The effect of these posttranslational modifications on the functions of these CCM proteins is unclear, as is the identity of the regulatory proteins upstream of these modifications. Methylation may also be involved in the regulation of pyrenoid biogenesis, as suggested by altered pyrenoid morphologies under low CO_2_ in mutants lacking function for the putative methyltransferase CIA6 (Cre10.g437829) (Ma et al. 2011).

When cells are transferred from limiting CO_2_ to high CO_2_, the disassembly of the pyrenoid is much slower than its assembly when cells are transferred from high CO_2_ to limiting CO_2_ (Kuchitsu et al. 1988; Ramazanov et al. 1994). The degradation of the starch sheath and the dissolution of the matrix can take two to three days (Ramazanov et al. 1994). This slow degradation is consistent with the slow deactivation of the CCM, which also requires about three days when cells are moved from limiting CO_2_ to high CO_2_ (Ramazanov et al. 1994). It has been suggested that CCM proteins are not rapidly degraded after the transition from low CO_2_ to high CO_2_ (Toguri et al. 1989), whereas the synthesis of new CCM proteins stops shortly after this transition (Manuel and Moroney 1988). These observations have not been confirmed for crucial pyrenoid proteins, and the regulatory mechanisms remain unclear.

### The pyrenoid-based CCM is induced and deactivated during the diurnal cycle

Most studies of the Chlamydomonas pyrenoid and CCM have been performed with asynchronous cultures of cells grown under constant light, where the induction of the CCM and formation of the pyrenoid are solely determined by the level of CO_2_. However, synchronous growth under diurnal cycles has revealed that the CCM is regulated during the course of the day/night cycle (Mitchell et al. 2014; Tirumani et al. 2014; Zones et al. 2015; Strenkert et al. 2019).

Chlamydomonas cells can be synchronized when grown under 12-h-light/12-h-dark cycles in minimal medium, with cells going through one cell cycle each day (Harris 2009; Mitchell et al. 2014; Zones et al. 2015; Strenkert et al. 2019). The CCM is downregulated at night and fully induced one hour before dawn (Mitchell et al. 2014). During this induction, both Rubisco and CAH3 were found to relocalize from the chloroplast to the pyrenoid based on statistical analyses of immunogold labeling (Mitchell et al. 2014), although it should be noted that the original electron micrographs were not provided in this study. Transcriptomics studies have shown that, in cells grown under diurnal cycles, genes encoding the master regulator CIA5 and crucial pyrenoid proteins reach their highest transcript levels in the first few hours around the transition from dark to light (Strenkert et al. 2019; Adler et al. 2022). However, whether CIA5 is also involved in CCM activation and pyrenoid formation during diurnal cycles is not known. The pyrenoid may grow and expand during the day as cells grow (Zones et al. 2015; Strenkert et al. 2019), but this has not yet been specifically measured.

### Pyrenoid formation can be induced by hyperoxia and H_2_O_2_

Hyperoxia was recently reported to induce pyrenoid formation, even at high CO_2_ or HCO_3_^−^ levels (Neofotis et al. 2021). The authors reasoned that because pyrenoid formation is induced by both low CO_2_ and hyperoxia, a metabolite that accumulates under both conditions may serve as a signal that induces pyrenoid formation. Consistent with this idea, the authors observed that hydrogen peroxide (H_2_O_2_), which is expected to accumulate under both conditions, induces pyrenoid formation. H_2_O_2_ is a byproduct of photorespiration, which recycles 2-phosphoglycolate, the toxic product of the oxygenase activity of Rubisco (Moroney et al. 2013), which is increased under low CO_2_ and hyperoxia. Whether H_2_O_2_ regulates pyrenoid formation directly or indirectly (e.g. via other metabolites) has not been determined.

### The Chlamydomonas pyrenoid matrix divides by fission and dissolves into the surrounding chloroplast during cell division

**Figure 5.**
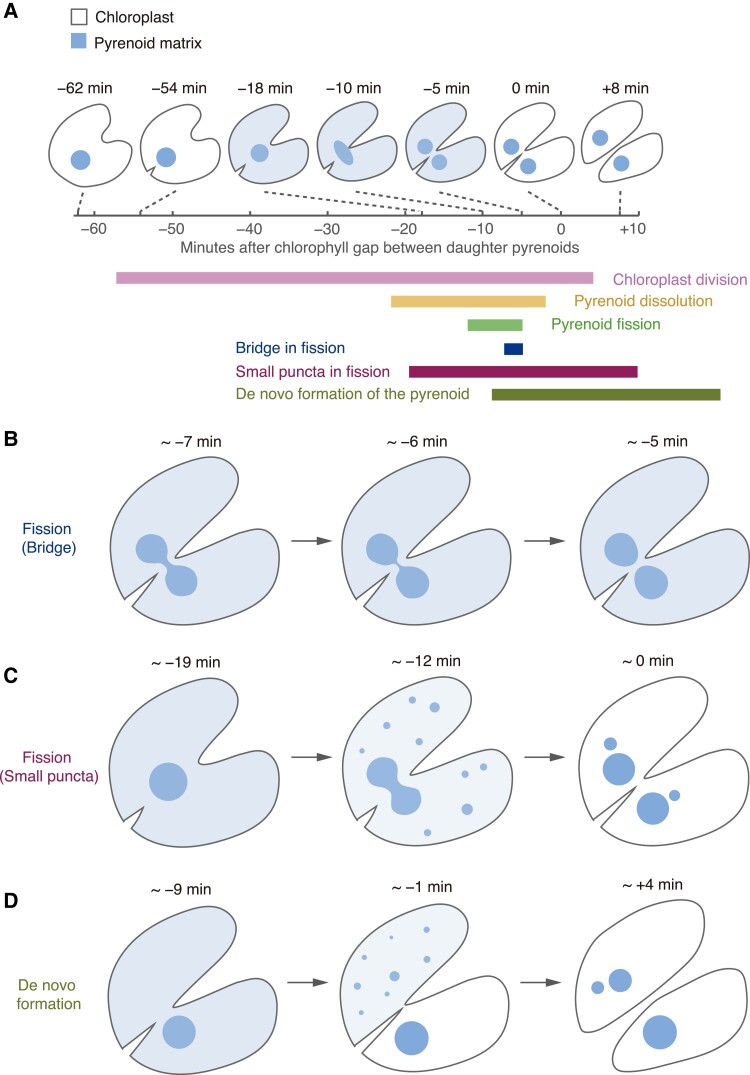
The Chlamydomonas pyrenoid exhibits liquid-like behavior during cell divisions. **A)** Diagram depicting the timeline and morphology of a typical cell division with pyrenoid fission in Chlamydomonas (adapted from Freeman Rosenzweig et al. 2017). The time point *t* = 0 is the moment the chloroplast division furrow passes between the daughter pyrenoids. A portion of the pyrenoid matrix disperses into the chloroplast stroma during the division of the pyrenoid. The approximate timing and duration of key events are shown below the timeline. **B)** Diagram depicting the “bridge” of matrix during pyrenoid fission. **C)** Diagram depicting the transient appearance of small puncta of pyrenoid matrix throughout the stroma during dispersal of the matrix in some dividing cells. **D)** Diagram depicting the de novo formation of a daughter pyrenoid when pyrenoid fission fails. The lower daughter cell inherits the entire pyrenoid of the mother cell. The upper cell shows de novo pyrenoid formation with the appearance of one or more fluorescent puncta growing or coalescing into one pyrenoid (observed in wild-type cells expressing either EPYC1-Venus or Rubisco-Venus).

Early electron microscopy studies observed the Chlamydomonas pyrenoid dividing by fission (Goodenough 1970). Recent live-cell microscopy observation of pyrenoid matrix dynamics using fluorescently tagged Rubisco or EPYC1 revealed that the matrix is inherited by fission in most chloroplasts and assembled de novo in others (Fig. 5) (Freeman Rosenzweig et al. 2017). Approximately two-thirds of daughter chloroplasts inherited their matrix through elongation and then fission of the pyrenoid matrix from the mother chloroplast (Fig. 5, A to C), whereas one of the daughter chloroplasts inherited the entire matrix punctum in the remaining cases (Fig. 5D). When the pyrenoid divided by fission, matrix elongation and fission occurred toward the end of overall chloroplast division and took approximately seven minutes. A “bridge” of matrix connecting the two lobes was briefly visible towards the end of fission (Fig. 5B). After the bridge ruptured, the daughter pyrenoids quickly reverted to spherical shapes, similar to the behavior of liquid droplets (Fig. 5B) (Stone 1994; Yanashima et al. 2012; Freeman Rosenzweig et al. 2017). The mechanism mediating pyrenoid fission during cell division is unknown.

A portion of the pyrenoid matrix rapidly disperses into the stroma approximately 20 minutes before pyrenoid division (Fig. 5C, at approximately 19 minutes, the light blue throughout the chloroplast indicates the dispersed pyrenoid matrix). During this dispersal, small puncta of matrix often transiently appear throughout the stroma (Fig. 5C). The dispersal of matrix materials may facilitate equal distribution of the pyrenoid matrix to daughter chloroplasts and may also help decrease the surface tension or viscosity of the matrix droplet to facilitate fission (Freeman Rosenzweig et al. 2017). Indeed, many of the daughter chloroplasts that did not inherit a pyrenoid through fission inherited dissolved matrix building blocks, from which they appeared to form a pyrenoid de novo (Freeman Rosenzweig et al. 2017) (Fig. 5D). In such cases, multiple small Rubisco or EPYC1 fluorescent puncta appeared, and smaller puncta shrank whereas larger ones grew until the cell contained a single pyrenoid (Freeman Rosenzweig et al. 2017) (Fig. 5D). This behavior resembles Ostwald ripening, a physical mechanism by which larger droplets in a phase-separated system grow by acquiring building blocks from smaller droplets (Hyman et al. 2014; Freeman Rosenzweig et al. 2017; Rosowski et al. 2020). The mechanisms regulating the formation of multiple puncta of matrix material and the dispersal of the matrix remain unknown.

Pyrenoids in other algae are likely to leverage liquid-like properties during cell division in ways similar to those observed in Chlamydomonas (Freeman Rosenzweig et al. 2017; Barrett et al. 2021). Indeed, both division by fission and the rapid disappearance and reappearance of the pyrenoid matrix (which would be consistent with matrix dissolution and condensation) have been observed during cell division using TEM on fixed cells in some species of the green algae *Tetracystis*, *Chlorococcum*, and *Bulbochaete* and the diatom *Donkinia* (Brown et al. 1967; Retallack and Butler 1970; Cox 1981). Additional discussions on liquid-like pyrenoid behavior during cell division can be found in the recent review by Barrett et al. (Barrett et al. 2021). Further studies on other species will be necessary to test the generality of this principle.

### The number of pyrenoids per cell appears to be regulated in Chlamydomonas

Wild-type Chlamydomonas cells have only one pyrenoid. However, in mutant cells lacking SAGA1, EPYC1, or CIA6, multiple pyrenoids are often observed, suggesting that wild-type cells may actively work to ensure that they have a single pyrenoid, and aspects of this regulation may be disrupted in these mutants.

The most striking example of multiple pyrenoids can be seen in the *saga1* mutant, with an average of approximately ten matrix droplets per cell (Itakura et al. 2019). The multiple pyrenoids in *saga1* are stable without obvious changes in size or position in living mutant cells over the course of one hour. Although the molecular function of SAGA1 remains unclear, these observations suggest that this protein is involved in maintaining a single pyrenoid.

The two other known cases of mutants with multiple pyrenoids are *epyc1* and *cia6*. Multiple pyrenoids were observed by TEM in 13% of *epyc1* cells compared with 3% in wild-type cells (Mackinder et al. 2016). A mutant of the putative methyltransferase CIA6 also exhibits multiple small pyrenoids, as observed by TEM or via light microscopy (Ma et al. 2011). TEM images showed that the morphology of the pyrenoids in *epyc1* and *cia6* are similar, with a small matrix and thicker starch sheath than in wild-type cells (Ma et al. 2011; Mackinder et al. 2016). EPYC1 has a well-characterized function in pyrenoid matrix biogenesis; thus, it is possible that defects in matrix biogenesis lead to multiple pyrenoids. The mechanism by which the number of pyrenoids in a cell is regulated remains to be identified.

## Perspective

The pyrenoid provides a unique opportunity to expand our fundamental knowledge of liquid-liquid phase separation, genetically engineer photosynthetic organisms for higher crop yields, and obtain insights into critical photosynthetic carbon assimilation in the oceans and fresh water, all through the study of one organelle.

Studies of the Chlamydomonas pyrenoid have laid the foundation for exploring the molecular composition of pyrenoids across the photosynthetic tree of life. Of particular interest is investigating whether pyrenoids in other species also exhibit liquid-like behavior. Additionally, exploring whether Rubisco-binding motifs are a common principle across all algal pyrenoids could lead to a better understanding of how pyrenoids first evolved. Molecular studies of CCM function in different algae could also help reveal the minimal components that will be necessary to create a functional pyrenoid in plants. More broadly, studying the mechanisms by which Rubisco condensates associate with membrane structures and peripheral polysaccharides could provide insights into general principles by which liquid-like organelles interact with other cellular structures.

Future studies in Chlamydomonas as well as in other species of algae will bring us closer to generating the first functional pyrenoid in vascular land plants, which will help us meet increasing agricultural demands as the global population rises. Progress is already being made toward engineering a Chlamydomonas-based pyrenoid into land plants with the successful reconstitution of Rubisco-EPYC1 condensates in Arabidopsis (Atkinson et al. 2020). The generation of thylakoid tubules that traverse these condensates, delivery of CO_2_ via these tubules, and the assembly of starch around these condensates will be important next steps.

In addition, by studying the regulation of pyrenoid formation and dynamics in Chlamydomonas as well as in dominant marine species such as diatoms, we can advance our understanding of the aquatic photosynthesis that is crucial for our ecosystem and the global carbon cycle.
